# Multivariate Interaction Analysis of Winter Wheat Grown in Environment of Limited Soil Conditions

**DOI:** 10.3390/plants10030604

**Published:** 2021-03-23

**Authors:** Nataša Ljubičić, Vera Popović, Vladimir Ćirić, Marko Kostić, Bojana Ivošević, Dragana Popović, Miloš Pandžić, Seddiq El Musafah, Snežana Janković

**Affiliations:** 1 Biosense Institute, University of Novi Sad, 21000 Novi Sad, Serbia; ljubicic.natasa@gmail.com (N.L.); bojana.ivosevic@biosense.rs (B.I.); milos.pandzic@biosense.rs (M.P.); 2 Institute of Field and Vegetable Crops, 21000 Novi Sad, Serbia; 3 Faculty of Agriculture, University of Novi Sad, 21000 Novi Sad, Serbia; vladimir.ciric@polj.uns.ac.rs (V.Ć.); marko.kostic@polj.uns.ac.rs (M.K.); 4 Faculty of Economics in Subotica, University of Novi Sad, 21000 Novi Sad, Serbia; draaaganap@gmail.com; 5 Institute of Applied Ecology Futura, University of Metropolitan, 21000 Belgrade, Serbia; seddiq.1975@gmail.com; 6 Institute of Applied Sciences in Agriculture, 21000 Belgrade, Serbia; sjankovic@ipn.co.rs

**Keywords:** wheat, genotype by environment interaction, solonetz soil, AMMI

## Abstract

The less productive soils present one of the major problems in wheat production. Because of unfavorable conditions, halomorphic soils could be intensively utilized using ameliorative measures and by selecting suitable stress tolerant wheat genotypes. This study examined the responses of ten winter wheat cultivars on stressful conditions of halomorphic soil, solonetz type in Banat, Serbia. The wheat genotypes were grown in field trails of control and treatments with two soil amelioration levels using phosphor gypsum, in amounts of 25 and 50 tha^−1^. Across two vegetation seasons, phenotypic variability and genotype by environment interaction (GEI) for yield traits of wheat were studied. The additive main effects and multiplicative interaction (AMMI) models were used to study the GEI. AMMI analyses revealed significant genotype and environmental effects, as well as GEI effect. Analysis of GEI using the IPCA (Interaction Principal Components) analysis showed a statistical significance of the first two main components, IPCA1 and IPCA2 for yield, which jointly explained 70% of GEI variation. First source of variation IPCA1 explained 41.15% of the GEI for the grain weight per plant and 78.54% for the harvest index. The results revealed that wheat genotypes responded differently to stressful conditions and ameliorative measures.

## 1. Introduction

Today, more than ever, great attention has been paid to agro-ecological conditions and issues of climate changes [1,2]. Constant striving to intensify agricultural production, excessive using of chemicals, fertilizers, as well as massive irrigation has led to degradation of arable land, pollution of the environment and significant climate change [3]. At the same time, the less productive soils present one of the major problems in agricultural production. Halomorphic soils, characterized as unfavorable soil for agricultural production, occupy significant areas of the world. The properties of halomorphic soils are controlled by the presence of either soluble salts, exchangeable sodium or both [4,5]. Soil degradation resulting from soil salinity and/or sodicity is recognized as a major problem of soil productivity and quality under arid and semiarid climates [6,7]. According to the current soil national classification, halomorphic soils are divided into a class of saline soils, characterized by high salt content and a class of alkaline soils characterized by a high content of adsorbed sodium in the second (Bt) horizon, which includes solonetz type of soil [8]. Worldwide, solonetz cover about 135 million hectares, with 20 million hectares in Europe. Major solonetz areas are in Ukraine, Russia, Hungary, Bulgaria, Rumania, China, Canada, less humid parts of South America, the south western and north-central part of the United States, South Africa and Australia [9]. In Vojvodina Province (North Serbia), beside of about a million hectares of high productive soils, there are about 80,000 ha less productive soils of halomorphic, solonetz soil type, mainly in Banat region [4]. These soils are most commonly used as extensive pasture and could be intensively utilized using ameliorative measures, which include incorporation of phosphogypsum in soil or by selecting suitable crops genotypes [10]. Long-term results indicated that the application of phosphogypsum in solonetz soil showed positive changes in the content and qualitative composition of cations, increases Ca^2+^ content in soil and decreases the alkaline reaction, thus improving the content and stability of the structural aggregates, water and air regimens, thermal and nutrient status, as well as the effective fertility of solonetz soils [11,12]. Phosphogypsum, produced by the phosphate fertilizer industry, as a by-product of wet acid production of phosphoric acid from rock phosphate, contains more than 92% calcium sulfate and presents suitable source of calcium, which may be used as a soil conditioner for sodic, solonets and solonetzic soils [13]. Nayak et al. [14] found that with the increasing amounts of phosphogypsum applied on agricultural soil without vegetation, pH was reduced from 7.9 in control to 5.1 in treatment with 20% phosphogypsum. Aside from its benefits, since phosphate ores have increased natural radioactivity, radionuclides are a special type of impurities that do not significantly affect the quality of phosphogypsum, but significantly affect the environment [15]. Hence, there is certain concern that the application of phosphogypsum to agricultural lands may results in plant uptake of radionuclides, fluoride and trace elements [16] Therefore, since phosphogypsum is formed as a by-product in the production of phosphoric acid in such large quantities, its storage is often an environmental problem [17]. However, agricultural production is greatly influenced by soil extremes and variability. The soil degradation process can significantly reduce plant diversity and agricultural yield, land productivity and value in arid and semiarid climate regions [18]. Adverse effects of high Na concentration in the B horizon on management practices and on seedbed preparation has been observed by Cairns [19], while adverse effect to the uptake of plant nutrients by the roots was observed by Peters [20] and Miller and Brierley [11]. Despite wheat being considered as basically grass, durable and more resistant to limiting factors in agricultural production than many other crops, the considerable decreases in yield and yield related traits have been observed in wheat production under stress conditions of halomorphic soils [5,21,22]. At the same time, global demand for wheat is estimated to increase by 2050, as a consequence of growing dietary requirements of an increasing world population [23]. Keeping this in view, the wheat breeders have to share an urgent need to increase wheat grain yield potential by developing new wheat varieties with desirable genetic make-up [24,25]. Considering that much more attention needs to be given not only to the quantity, one of the greatest challenges to wheat breeders is to obtain a wheat genotypes with high mean yield and wide adaptation to the various environments, even in less productive soils, such as a solonetz soil type. Wide adaptation is defined as the genotypes ability to produce relatively high yields consistently across diverse agricultural environments in a growing region [26,27,28]. An assessment of the stability of cultivars yield provides valuable information about their behavior in specific environments [29,30]. The complexity of grain yield and yield related traits is the result of different genotype reactions to changing environmental conditions during plant development [30]. Grain yield is a complex polygenic, quantitative trait, whose expression is the result of the genotype, environment and genotype by environment interaction (GEI). GEI reflects the different responses of the genotypes to environment conditions and distinctly indicates that the best genotypes in a specific environment could not be the best for others. Hence, GEI cannot represent all genetic potential environmental conditions, which makes difficult the recommendation of genotypes by the breeder [31,32,33]. For accessing and better understanding the GEI effects, several statistical methods have been developed. The additive main effects and multiplicative interaction (AMMI) model has been one of the most widely used statistical tools in the analysis of GEI [30,34,35,36,37]. The AMMI model combines firstly the analysis of variance (ANOVA) to partition the variation into genotype main effects (G), environment main effects (E) and genotype by environment interaction (GEI) effects and then it applies principal components analysis (PCA) to GEI in a single analysis [37]. The two main purposes of AMMI analysis of a yield trial’s treatment design are: (i) Understanding complex GEI, which includes delineating mega-environments and selecting genotypes to exploit narrow adaptations; and (ii) increasing accuracy to improve recommendations, repeatability, selections and genetic gains 1 [33]. Therefore, accurate determination of the source of variation, their effects on yield and yield related traits and selection wheat genotypes that have been adapted to limited conditions of solonetz soil is of great importance. In this respect, the aims of the present research were: (i) To determine the influence of a genotype, environment and their interaction on grain yield and yield related traits in different wheat varieties; and (ii) to evaluate stability of the traits using the AMMI model in conditions of different levels of melioration on solonetz soil type. These results could provide the identification of suitable and stable wheat genotypes, which can be successfully used in wheat production on less productive, solonetz soils.

## 2. Materials and Methods

### 2.1. Field Trial

The present study was carried out at the experimental trial field on halomorphic soil of solonetz on location Kumane (45.539° N, 20.228° E), in Banat region, in Vojvodina Province (Serbia), during two consecutive vegetation seasons of 2004/2005 and 2005/2006. The experimental material in the study was comprised of ten winter wheat cultivars (*Triticum aestivum* L.), namely, Mina (G1), Sofija (G2), Tiha (G3), Anastazija (G4), Nevesinjka (G5), Evropa 90 (G6), NSR-5 (G7), Dragana (G8), Ljiljana (G9) and Simonida (G10). The wheat cultivars used in the study were released by the Institute of Field and Vegetable Crops, Novi Sad, in Serbia. All cultivars are agronomically suitable for production in agroecological conditions of Serbia and other surrounding countries. The experimental trial was set up on solonetz type of soil, according to the completely randomized block design (RBCD), with three amelioration treatment and three replications of each treatment. The experimental plots consisted of eight 12.5 cm-spaced rows, 2 m in length. Plant spacing was realized by implementing a plant density of 500 seeds per m^−2^. Disease, weed and pest control on each plot was performed by using standard cultivation practice, while the NPK (15:15:15) application was split, 50 kg for each treatment. Planting in both growing seasons was completed by the second decade of October, while harvest was ended in the first decade of July. Since the experiment was set on solonetz type of soil, in addition to the results that were analyzed were the results of the soil in two levels maintenance, being 25 and 50 tha^−1^ phosphogypsum. The objective of applying reclamation measures to solonetz soils is to change their cation content which makes changes in chemical reactions, because increasing the calcium cation level and reducing the alkaline reaction improves the stability of structural aggregates, thermal, water, air properties and nutrient status [38]. The first treatment in the trial was soil without amelioration (control), the second treatment was amelioration using phosphor-gypsum in the amount of 25 tha^−1^ and the third treatment was amelioration using phosphor-gypsum in the amount of 50 tha^−1^. In the field trial, each treatment in one growing season was considered as a special agro-environment. This produced six different agro-ecological environments, which were equal in agrotechnical terms, but in different treatments of phosphogypsum melioration of soil repair. The six analyzed environments were labeled as follows: E1 represents control—solonetz soil without melioration in the season 2004/2005, E2 represents solonetz soil with melio-ration of 25 tha^−1^ of phosphogypsum in the season 2004/2005, E3 represents solonetz soil with melioration of 50 tha^−1^ of phosphogypsum in the season 2004/2005, E4 represents control—solonetz soil without melioration in the season 2005/2006, E5 represents solonetz soil with melioration of 25 tha^−1^ of phospho gypsum in the season 2005/2006, E6 represents solonetz soil with melioration of 50 tha^−1^ of phosphor gypsum in the season 2005/2006, Table 1.

At the stage of full maturity, ten average wheat plants from each replication of each wheat cultivar plot separately were selected and yield traits, such as a plant height (cm), grain weight per plant (g) and harvest index (%), were analyzed. The main sample consisted of 10 plants per replication, while grain yield, expressed in m^−2^, at 13% moisture, were obtained by harvesting each wheat cultivar plot separately.

### 2.2. Soil Properties

The experiment was set up at the of halomorphic soil, solonetz type in Banat, Serbia. This type of soil is characterized by unfavorable physical and chemical properties, caused by high content of clay and sodium in the Bt,na horizon. The presence of adsorbed sodium in the Bt horizon causes peptization of colloids and a high alkaline reaction of soil [38]. Table 2 shows the soil properties until a depth of 60 cm of typical solonetz, from which soil samples were taken to establish the experiment.

Solonetz soil is characterized by a lower concentration of salt and possesses a weakly acidic reaction which, with increasing depth, turns into an alkaline reaction. The content of CaCO_3_ is missing in the soil surface layer and its content increases with depth of soil. The content of humus and nitrogen in the surface layer is high, but drastically decreases with increasing depth. The soil is poor in phosphorus, while the potassium content is optimal. Permeability of solonetz soil is reduced and water penetration into the deeper horizons is possible only through the soil cracks in the dry period [39]. The solonetz type of soil is unsuitable for agricultural production due to the poor water and air regime, the possibility of stagnation and compaction during dry periods.

### 2.3. Meteorological Conditions

**Figure 1:**
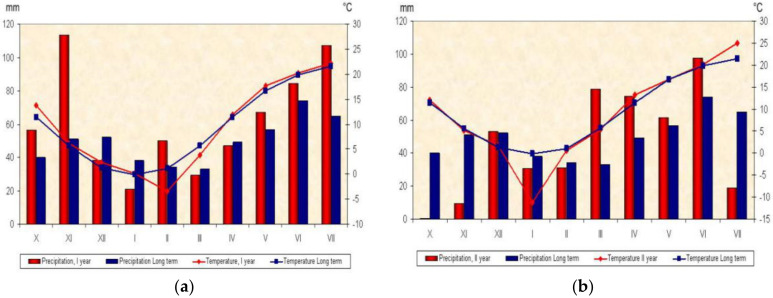
Weather characteristics of the vegetative seasons: Average monthly air temperature (°C) and precipitation (mm) and perennial average for locations in the first year (**a**); average monthly air temperature (°C) and precipitation (mm) and perennial average for locations in the second year (**b**).

The meteorological conditions of vegetation seasons during the trial were obtained from automatic weather stations of the Republic Hydro-meteorological service of Serbia, situated in Bečej, near to the field trials. The weather conditions in the first vegetation season of 2004/2005 were more favorable for wheat cultivation than climatic conditions of 2005/2006 growing season, Figure 1a,b.

The most significant characteristic of weather conditions in the first vegetation season was unusually high amounts of precipitation. Climatic conditions for wheat growth and development in this period can be characterized as good. Wheat planting was done in the optimal time and plants entered prepared for the conditions of the winter period. Although the winter was cold, the snow cover protected the winter crops from frosts and provided excellent thermal insulation to the crops. At the end of the winter, partial melting of snow lead to an increase in soil moisture reserves. The spring frosts did not cause damage. May and June were characterized by average weather conditions.

Climatic conditions in the second vegetation season were significantly more variable compared to the conditions in the previous season. The second vegetation season had unusually frequent very large and rapid changes in the values of meteorological elements that most affect the plant growth and wheat development stages. On several occasions, there was a change of periods with extremely cold and extremely warm weather, periods with floods and droughts whose length could be measured in weeks or months. Wheat planting was done in the optimal time, but at the time of sowing, due to the deficit of precipitation, there was a strong soil drought and wheat germination was difficult. November saw an extreme precipitation deficit, in relation to the multi-year average. During the winter, there were extremely low temperatures and long-lasting frosts which were unfavorable for wheat due to the lack of snow cover. Late winter and early spring were characterized by heavy rainfall, floods and long-term wetlands. April, May and the middle of June were significantly colder with high precipitation, while in the second half of June, the average daily air temperatures significantly exceeded the multi-year average value. It was characterized by extreme heat and critical wheat phases in the grain development took place in extreme drought conditions. Air temperature values were generally higher than optimal at the time of grain filling, while soil moisture reserves were rapidly depleted due to intensive evapotranspiration, Figure 1a,b.

### 2.4. Statistical Analyses

Genotype by environment interaction (GEI) was estimated using AMMI (additive main effects and multiplicative interaction) analysis developed by Zobel et al. [33]. The AMMI model Zobel et al. [33] is presented as the following formula:

Y_ger_ = μ + α_g_ + β_e_ + Σλ_n_ξ_gn_η_en_ + Θ_ge_ + ε_ger,_ (1)

where y_ge_ is the mean of yield or other observed trait for genotype *g* in the environment *e*, μ—grand mean, α_g_—genotypic mean deviations, β_e_—environmental mean deviations, n—number of PCA axis retained in the adjusted model, λ_n_—eigenvalue of the PCA axis *n*, ξ_gn_—genotype score for PCA axis n, η_en_—score eigenvector for PCA axis n, Θ_ge_—residual and ε_ger_—experimental error.

The AMMI model incorporates analysis of variance (ANOVA) and principal components analysis (PCA) into a single statistical model [40]. In the AMMI model, the ANOVA separate additive effects from the interaction, while additional GEI analysis can be carried out by principle component analysis (PCA). The biplot graphic representation shows both the main and interaction effects for genotypes and environments simultaneously and provides analysis of the G × E interaction [22,36,40,41]. The IPCA1 score of a genotype in the AMMI analysis was used as an indicator of the stability of a genotype over environments [12,42]. Zero IPCA value indicates highest stability, while an IPCA value long distance from zero indicates genotype instability [43,44]. The data processing was performed in GenStat 9th Edition statistical software package (trial version) VSN International Ltd. (www.vsn-intl.com, accessed on 11 December 2020). [45].

## 3. Results and Discussion

Grain yield is a complex super trait consisting of many individual yield components and largely dependent on the genetic potential, which considerably varies primarily as a result of environmental conditions during the growing season [36,46,47]. Changes in certain morphological traits and yield stability have led to an increase in the genetic potential of the genotypes, most probably through the increased tolerance to biotic and abiotic stress factors [48]. Contribution of each individual yield component could vary in the different environmental conditions; testing in one environment could provide only limited information, while testing in different environments provides additional useful information (e.g., a GEI component can be estimated) [49]. Since that interaction represents the heterogeneity of agro-ecological conditions, it is important to identify genotypes that are very efficient, which means that they are productive and stable genotypes, but also genotypes with favorable response to soil amelioration. Estimation of GEI may prove valuable in recommending suitable wheat genotypes, as well as change of agricultural practice.

### 3.1. Plant Height

The presented results showed that the mean values of plant height of observed wheat genotypes in the trial ranged between 62.1 (E3) and 63.5 cm (E1 and E2) within different environments in the first vegetation season. In the second vegetation season, the mean values of plant height of wheat ranged between 42.8 (E4) and 58.6 cm (E5) within different environments. Between genotypes, the mean values of plant height ranged from 52.4 (G3) to 61.5 cm (G6) within different genotypes (Table 3). In the first vegetation season, at the control variant (E1), solonetz with no amelioration applied, the greatest overall mean values for plant height were denoted for wheat varieties G6 (68.6 cm), G4 (66.9 cm) and G5 (65.6 cm). At the same variant, the lowest mean values of the plant height were observed for genotypes G10 (59.3 cm), G2 (59.9 cm) and G3 (60.2 cm). In the second vegetation season, at the control variant (E4), the greatest overall mean values were denoted for genotypes G6 (47.8 cm), G5 (47.1 cm) and G4 (47.0 cm). The lowest values of plant height were denoted for wheat genotypes G10 (35.6 cm), G2 (38.7 cm), G1 (40.1 cm) and G3 (40.1 cm), Table 3.

In the second environment, with soil amelioration of 25 tha^−1^ phosphor gypsum applied, during the first vegetation season (E2), the greatest overall mean values for plant height were denoted for wheat genotypes G6 (68.5 cm), G4 (66.4 cm), G8 (65.2 cm) and G7 (64.9 cm). The lowest values of stem height were denoted for wheat genotypes G3 (59.8 cm), G2 (60.0 cm) and G10 (60.9 cm), Table 3.

In the third environment during the first vegetation season at the amelioration of 50 tha^−1^ phosphor gypsum applied (E3), the greatest overall mean values for plant height were observed for wheat varieties G6 (67.1 cm), G8 (64.7 cm), G4 (64.5 cm) and G7 (63.0 cm), while the smallest values showed for genotypes G3 (57.9 cm), G2 (58.9 cm) and G1 (60.3 cm). In the second vegetation season, with same level of amelioration (E6), the greatest overall mean values of grain yield were denoted for wheat varieties G6 (53.27 cm), G8 (52.63 cm), G10 (51.85 cm) and G4 (49.47 cm), while the smallest values showed for G3 (43.18 cm), G5 (43.99 cm) and G2 (45.69 cm), Table 3.

The results of this study showed that significant plant height variation was noticed due to different environment conditions, but also depended on genotype, as well as on vegetation season. This result is expected since plant height is one of the important yield components and is considered as a quantitative and variable trait whose expression highly depends on the environmental factors [36,50,51]. This was confirmed by high values of variation which ranged from 39.6% to 86.8% (Table 3).

According to the results, a higher sensitivity of plant height to the meteorological conditions was observed in the second vegetation season, since the overall plant height average was the lowest in the control variant. On the other side, more favorable conditions in the first growing season exhibited a more significant impact on reducing the differences between the mean averages per treatment. In terms of the second vegetation season, higher sensitivity of plant height of wheat was mostly caused by drought conditions after planting, winter frost and a less amount of rainfall in the first part of the growing season, and the effects of ameliorative measures were the most evident.

Apart from climatic conditions, tested wheat genotypes on solonetz soil reacted differently on different levels of soil repair. The phenotypic expressions of plant height of certain genotypes were constant in various environments, whereas some other wheat genotypes exposed significant variation over different soil amelioration treatments. In this sense, genotypes G2, G8 and G10 expressed a higher sensitivity to the treatment and reacted favorably on ameliorative measures from 25 (G2 and G8) to 50 tha^−1^ (G10) in the first season, while in the second season all tested genotypes reacted favorably on both amounts of applied measures. In general, vegetation periods of 2004/2005 provided generally higher mean values of the plant height than vegetation periods of 2005/2006. Varieties responded to ameliorated measures enhancing the plant height in favorable agro-ecological conditions. In addition, more favorable weather conditions of the first vegetation season exhibited significant impact on reducing the differences between the averages per treatment. Hence, the changing weather conditions influenced the effect of amelioration on the soil and the observed variation in the plant height of wheat.

The AMMI analysis of variance showed significant effects for genotypes and environments (Table 4).

The combined ANOVA showed that the phenotypic expression of plant height was significantly influenced by environmental variations, because the significant variance at the 1% level explained 80.14% of the total variation, while genotype contributed 8.64% of the total variation of the experiment (Table 4).

A large sum of squares for the environments indicated that the environments were diverse, with large differences among environmental means causing variation in the plant height. Differences between the growing seasons and diversity of treatments caused a considerable sum of squares for environmental factors in the total variation, indicating that these factors were the most responsible for the variation of the plant height of wheat. GEI contributed 11.23% of the total sum of squares and expressed no significant mean square, indicating that no cross interaction was expressed for the plant height. Large differences among environments and vegetation seasons caused a high sum of square of environmental factors in the overall variation of the trial, and led to the conclusion that they are the most responsible for variations in plant height of wheat. Such results are in agreement with results reported by Popović et al. [36,52] as well as with results by Branković-Radojčić et al. [53], who stated that, in multi-environment trials, often environments explain about 80% of the total variation, while the genotype (G) and GEI share is about 10%. This result was expected since the plant height of wheat is considered as a quantitative and highly variable trait whose expression highly depends on the environmental factors [36,54]. Additional analysis of the GEI using the PCA (interaction principal components) analysis confirmed there were no statistical significance of the first two principal components, IPCA 1 and IPCA 2 (Table 4). Separately, IPCA1 and IPCA2 participated in the GEI variation, with 36.6% and 29.1%, respectively. Both without a statistically significant effect on the GE interaction variation. These two main components explained, jointly, 65.6% of the variation of the genotype by environment interaction (Table 4).

**Figure 2:**
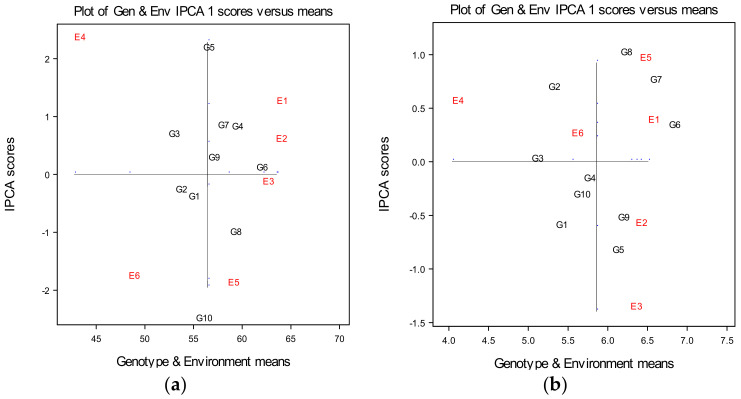
(**a**) AMMI 1 biplot of ten wheat cultivars across six environments (two years × three treatments) for the estimation of main and multivariate (GEI) effects for the plant height of wheat (cm); (**b**) AMMI 1 biplot of ten wheat cultivars across six environments (two years × three treatments) for the estimation of main and multivariate (GEI) effects for the grain weight per plant of wheat (g).

In the AMMI biplot, the genotype and environment main effects for plant height of ten winter wheat genotypes are presented on the x-axis, while the IPCA1 (interaction principal component axis 1) scores are on the y-axis. The vertical line is the grand mean for trait plant height and the horizontal line (y-ordinate) represents the IPCA1 value of zero (Figure 2a). Observing trends in PCA1 scores and based on trends in two-dimensional graphical representation of genotypes and environments, wheat varieties were not differing in relation to main effect, but certain differences could be denoted in multivariate effect.

According to the biplot (Figure 2a) and in terms of average values, it could be noticed that agro-ecological environment E3 was at the level of the experimental overall average. Small distance environmental points from the origin (zero point) were also denoted for environment E2, which indicated that environments E2 and E3 were assessed as the most favorable to achieve a stable reaction to plant height. Environment E1, which correspondent to control variant, was further from the origin, while environments E4, E5 and E6 had the highest interaction values and contributed a lot to the GEI, which determines them the least suitable for stable establishment plant height of wheat. According to the arrangement of environmental points E1, E2, E3 and E5 in relation to the mean value of trait, it can be concluded that the genotypes achieved higher average values of plant height in these environments compared to E4 and E6 points. This result does not favor the E5 environment, as well as E4 and E6, for obtaining higher values of plant height, given the high values of interaction indicate the poor stability of this trait. In general, distribution environment points on the graph revealed that the more stable reaction was achieved in the first vegetation season (Figure 2a).

Based on the graphic presentation of the interaction of genotypes and environments, ten winter wheat genotypes differed in genotype by environment interaction. Wheat genotype G6 (Evropa) expressed the most stable reaction, showing a PCA1 score quite near to 0. Genotypes G1, G2, G3 and G9 showed values close to zero and they had small interaction effects, small contribution to the GE interaction and fitted well into the additive model. Genotypes G4, G7 and G9 showed higher distance from origin, while genotypes G10 and G5 were furthest from the PCA axis, thus they proved to be the most unstable and contributed a lot to the GE interaction captured by the first axis IPCA1. These two genotypes showed the highest interaction at the level of the whole experiment. Genotype G6 appeared to be better adapted to the E3 and E2 environments, which corresponded to soil treatments of 25 and 50 tha^−1^ phosphogypsum, having a mean value of plant height above the overall mean. Genotypes G9, G7 and G4 expressed also a positive effect in E3 and E2 environments, which correspond to environments with 25 and 50 tha^−1^ phosphogypsum applied, keeping their average quite above the level of overall mean. Genotype G8 appeared to be better adapted to conditions of E3 and E5, which corresponded to soil treatments of 25 and 50 tha^−1^ phosphogypsum, while genotypes G2 and G1 were better adapted to conditions of E6, which corresponded to soil treatment of 50 tha^−1^ phosphogypsum. Genotype G3 appeared to be adapted to conditions of E2, E3 and E1 environments, keeping its average below the overall mean (Figure 2a). According to Gauch [32], the genotypes at the top of the graph table have positive G × E interactions with environments and those genotypes at the bottom have negative G × E interactions with environments. Therefore, genotypes at the top of graph (G3, G4, G5, G6, G7 and G9) showed positive G × E interactions with environments, while genotypes which were at the bottom (G1, G2, G8 and G10) showed negative G × E interactions with environments (Figure 2a).

Obtained results are in accordance with results of AMMI selection of wheat genotypes across the testing environments (Table 5). 

Based on the tested genotypes, genotype G6 could be considered as the most promising, since it ranked within the first four AMMI selections in all environments and showed consistent performance, implying it having the best adaptability for all soil conditions. In soil environment E1 (control, soil without amelioration) in the first vegetation season, genotype G6 was first ranked, followed by genotypes G4, G8 and G7. In soil environment E4 (control, soil without amelioration) in the second vegetation season, genotype G6 was first ranked, followed by genotypes G4, G5 and G7. This result indicates that these genotypes have a positive response and stable reaction in less favorable conditions in the control environment. In soil environment E2, which presents soil treatment with 25 tha^−1^ phosphogypsum in the first season, genotype G6 was first ranked, followed by genotypes G8, G4 and G7. In soil environment E5 (treatment with 25 tha^−1^ phosphogypsum) in the second season, genotype G6 was first ranked, followed by genotypes G8, G10 and G4. This indicates that these genotypes have positive response on the soil melioration treatment with 25 tha^−1^ phosphogypsum. In soil environment E3, which corresponds to soil treatment with 50 tha^−1^ phosphogypsum, in the first season, genotype G6 was first ranked, followed by genotypes G5, G4 and G7. In soil environment E6, which corresponds to soil treatment with 50 tha^−1^ phosphogypsum, in the second season, genotype G6 was first ranked, followed by genotypes G8, G10 and G4. The results confirm that it is possible to successfully advise the right genotype for all environments, or specific genotypes for specific environments, through AMMI evaluation (Table 5).

However, genotypes which maintain their height in stress environments could be desirable in certain limited conditions, because greater expression of plant height has been frequently attributed to high capacity of stems to accumulate sufficient stem reserves for the partitioning to grain.

### 3.2. Grain Weight per Plant

Grain weight as a quantitative trait, together with several yield related traits contributes to the formation of total grain yield of wheat. Each yield component is controlled by genes and affected by environmental factors, as well as by the interaction of genetic and environmental factors [55]. Grain weight per plant directly reflects the efficient use of nutrients and their translocation into generative parts of plant [56]. Grain weight per spike, as grain weight per plant, as the last yield components, are the final in the development of many components that occur in the early ontogenic stages [56].

The presented results showed that the mean values of the grain weight per plant of observed wheat genotypes ranged between 6.29 (E3) and 6.51 g (E1) within different environments in the first vegetation season. In the second vegetation season, the mean values of grain yield per plant of wheat ranged between 4.04 (E4) and 6.40 g (E5) within different environments. Between genotypes, the mean values of grain yield per plant ranged from 5.04 (G3) to 6.77 g (G6) within different genotypes (Table 6).

At the control variant (E1), with no amelioration applied, the greatest overall mean value for grain yield per plant was denoted for wheat variety G6 in both years of the study, having values of 7.53 g in the first season and 5.11 g in the second season. In both seasons the same trend was observed between genotypes and the higher values were observed for genotypes G7, G8 and G9. In the second environment (E2), during the first vegetation season at the amelioration of 25 tha^−1^ phosphor gypsum applied, the greatest overall mean values for grain weight per plant were denoted for wheat varieties G5 (7.09 g), G6 (7.08 g), G9 (6.97 g) and G7 (6.58 g). In the second season, with same level of amelioration (E5), the greatest overall mean values were denoted for wheat varieties G7 (7.74 g), G8 (7.61 g), G6 (7.59 g) and G2 (6.40 g), Table 7. In the third environment, during the first vegetation season at the amelioration of 50 tha^−1^ phosphor gypsum applied (E3), the greatest overall mean values for grain weight per plant were denoted for wheat varieties G6 (6.78 g), G7 (5.96 g), G8 (5.23 g) and G9 (7.35 g). In the second season, with the same level of amelioration (E6), the greatest overall mean values of grain weight per plant were denoted for wheat varieties G5 (5.56 g), G9 (5.70 g), G6 (6.53 g) and G1 (4.90 g). Presented results revealed that different treatments influenced the differences in grain weight per plant (Table 6).

According to the results, it was observed that the greater response of grain weight per plant were expressed in growing conditions of second vegetation season, due to the meteorological conditions, as the average value was the lowest in the control variant on solonetz without ameliorative measures. In this investigation, the temperature and precipitation regimes were different across years and each cultivar expressed a specific response to environmental conditions. More favorable conditions of the first vegetation season exhibited a more significant impact on minimizing the differences between the average values per treatment. During the second vegetation season, which began with extreme drought conditions after wheat planting, followed with long term frosts during winter without snow cover and unfavorable conditions during critical stages of grain development, the differences were increased and the effects of ameliorative measures were the most manifested (Table 6). According to Khan et al. [57] and Knežević et al. [55], high temperature shortens the grain filling period and induces early maturity, which causes grain shrinkage and low grain weight.

However, aside from climatic conditions, tested wheat genotypes on solonetz soil reacted differently on different levels of soil repair. The phenotypic expressions of grain weight per plant of certain genotypes were constant in various environments, whereas some other wheat genotypes exposed significant variation over different soil amelioration treatments. In this sense, genotypes G1, G4, G5, G9 and G10 expressed a higher sensitivity to the treatment and reacted favorably on both levels of soil ameliorative measures in the first season. While, in the second season, all tested genotypes reacted favorably on both amounts of applied measures. In general, the first vegetation period provided generally higher mean values of the grain weight per plant than the second vegetation period.

However, manifested differences due to years justify the establishment of multi-year trials to evaluate cultivars’ stability. When cultivars are evaluated across several years, the assessment is more reliable and cultivar means are characterized by a smaller error of estimation. These approaches provide support in evaluating cultivar adaptation and selecting cultivars with wide adaptation to environmental conditions [58,59].

The combined ANOVA showed that all three sources of variation (genotypes, treatments and environment) had a significant influence on phenotypic variation of trait grain yield per plant (Table 7). In the combined analysis of variance, the main effects, genotypes and environments were highly significant (52 + 136.1)/284.1 and explain 66.2% of total variation. Participation of the genotype variation in the treatments sum of squares (SS) amounted to 18.3%, while 48.9% of the total sum of squares was attributable to environmental effects (Table 8). A large sum of squares for the environments revealed that the trial environments were diverse, with large differences among environmental means, causing variation on grain yields per plant of wheat. This result is in accordance with results obtained by Rad et al. [51], Mohammadi et al. [42] and Popović et al. [36].

The genotype by environment interaction (GEI) expressed no significant mean square, leading to the conclusion that no cross interaction was expressed for the grain yield per plant of wheat. Furthermore, the additional analysis of the GEI using the PCA analysis confirmed the statistical significance of the first source of variation (i.e., the first main principal component IPCA1), which participated in the GEI variation with 41.15% (Table 7). Therefore, the interaction of the ten wheat genotypes within six environments was best predicted by the first interaction principal component of genotypes and environments. This result is expected, since that grain weight per plant, as well as, per spike, is a quantitative trait controlled by a number of minor genes and under the high influence of environmental conditions. Change in grain weight per plant drastically influences the final grain yield. A high share of the first principal component in the overall GEI, confirmed by AMMI analysis of grain yield, has been reported by Banjac et al. [12] and Popović et al. [36].

According to graphical representation of genotypes and environments in the biplot and in terms of average values (Table 6), a small distance of the environmental points from the origin (zero point) could be noticed, which indicates that the environments E6, E1 and E2 were assessed as the most favorable to achieve a stable response of grain weight per plant of wheat (Figure 2b). According to the arrangement of E1, E2, E3 and E5 points, it can be concluded that the wheat cultivars achieved higher average values of grain yield per plant in these environments compared to E4 and E6 points. This result does not favor the E3 and E5 environments for obtaining higher values of grain weight per plant, as the high values of interaction indicate the poor stability of this trait. Environments E3 and E5, as well as E4, had the highest interaction values, which determines them the least suitable for stable establishment grain weight per plant of wheat. Genotypes G3 and G4 were the most stable over all environments, indicating almost no cross interaction. Small distances of the genotypes points from the origin were also observed for genotypes G10, G6, G9 and G1. Genotypes with above average means, such as G6 and G9, could be selected based on grain weight per plant, while genotypes G8, G7 and G5 had a high distance from the average environment ordinate, thus showing them to be more variable and less stable across environments. Wheat variety G6 appeared to be better adapted to the less favorable conditions of the E1 control environment, showing good stability for this trait with small GEI and responded well, keeping its average above than overall mean. Varieties G2 and G8 responded favorably within treatments of 25 tha^−1^ phosphogypsum applied in the second growing season, while genotypes G9, G5, G1, G4 and G10 responded favorably within the same treatments applied in the first growing season, (Figure 1b). Genotypes G2 and G3 expressed a positive effect of interaction in E3, within soil treatment of 50 tha^−1^ phosphogypsum applied (Figure 1b). However, according to Gauch and Moran [32], genotypes at the top of graph (such as G2, G3, G6, G7 and G8) showed positive G × E interactions with environments, while genotypes which were at the bottom (such as a G1, G4, G5, G9 and G10) showed negative G × E interactions with environments (Figure 2b).

These results are in accordance with AMMI selection of wheat genotypes across the testing environments (Table 8). Results of the mean grain weight per plant of the environments, and their respective scores on the first axis of GEI, showed that genotype G6 could be considered as the most promising genotype, since it ranked within the first four AMMI selections in all environments. It showed consistent performance, implying its best adaptability for all soil conditions. In soil environment E1 (control, soil without amelioration) in the first vegetation season, genotype G6 was first ranked, followed by genotypes G7, G8 and G9. In soil environment E4 (control, soil without amelioration) in the second vegetation season, genotype G5 was first ranked, followed by genotypes G9, G6 and G1.

These results indicate that these genotypes have positive response and a stable reaction in less favorable conditions in control environments. In soil environment E2 (treatment with 25 tha^−1^ phosphogypsum) in the first vegetation season, genotype G5 was first ranked, followed by genotypes G9, G6 and G7. In soil environment E5 (treatment with 25 tha^−1^ phosphogypsum) in the second vegetation season, genotype G7 was first ranked, followed by genotypes G8, G6 and G2, which indicates that these genotypes have a positive response on soil melioration treatment with 25 tha^−1^ phosphogypsum. In soil environment E3 (treatment with 50 tha^−1^ phosphogypsum) in the first vegetation season, genotype G5 was first ranked, followed by genotypes G9, G6 and G1. In soil environment E6 (treatment with 50 tha^−1^ phosphogypsum) in the second vegetation season, genotype G6 was first ranked, followed by genotypes G7, G8 and G9, which indicates that these genotypes have a positive response on soil melioration treatment with 50 tha^−1^ phosphogypsum. The results, shown in Table 8, confirm that it is possible to successfully advise the right genotype for all environments, or specific genotypes for specific environments, through AMMI evaluation.

### 3.3. Grain Yield

The presented results showed that the mean values of grain yield of wheat, expressed in gm^−2^, ranged between 705.6 (E3) and 728.7 g (E1) within different environments in first vegetation season. In the second vegetation season, the mean values of grain yield of wheat ranged between 466.0 (E4) and 717.2 g (E5) within different environments. Between genotypes, the mean values of grain yield ranged from 564.70 (G3) to 760.70 g (G6) within different genotypes (Table 9). At the control variant (E1), with no amelioration applied, the greatest overall mean values for grain yield in first vegetation season were denoted for wheat varieties G8 (819.90 g), G5 (816.50 g) G4 (785.90 g) and G4 (0.54%). In the second vegetation season, at the control variant (E4), the greatest overall mean values were denoted for genotypes G6 (632.60 g), G7 (631.50 g), G9 (538.90 g) and G8 (535.80 g), Table 9. In the second environment, with soil amelioration of 25 tha^−1^ phosphor gypsum applied, during the first vegetation season (E2), the greatest overall mean values for grain yield were denoted for wheat varieties G5 (837.20 g), G6 (767.00 g), G4 (740.80 g) and G9 (736.90 g). In the second vegetation season, with the same level of amelioration (E5), the greatest overall mean values were denoted for wheat varieties G7 (889.40 g), G6 (865.30 g) and G8 (848.80 g), Table 9.

In the third environment during the first vegetation season at the amelioration of 50 tha^−1^ phosphor gypsum applied (E3), the greatest overall mean values for grain yield were denoted for wheat varieties G9 (863.50 g), G5 (822.30 g) and G6 (804.80 g). In the second vegetation season, with same level of amelioration (E6), the greatest overall mean values of grain yield were denoted for wheat varieties G6 (731.90 g), G7 (714.30 g) and G8 (675.40 g), Table 9.

Presented results revealed that different treatments influenced the differences in grain yield of wheat, but also depended on vegetation season. According to the results, a higher sensitivity of grain yield to the meteorological conditions were observed since the overall yield average was the lowest in the control variant in growing conditions of 2005/2006. It can be clearly seen from Table 9 that more favorable conditions in the growing season of 2004/2005 exhibited a more significant impact on reducing the differences between the mean averages per treatment. On the other side, in the second season of 2005/2006, which was characterized by less rainfall in May and stronger precipitation during June, the effects of ameliorative measures were the most evident. Higher sensitivity of grain yield in the second season was caused by high precipitation during flowering and further important developmental stages in wheat. Apart from climatic conditions, genotypes G1, G5, G6, G9 and G10 expressed a higher sensitivity to the treatment and reacted favorably on ameliorative measures in both seasons. Observed variations for grain yield found among these genotypes indicated the high genetic potential for further yield improvement. The similar findings and the influence of climatic conditions on grain yield of wheat has been reported by several authors [22,36].

The combined analysis of variance showed that all three sources of variation, genotypes, treatments and their interactions were highly significant and had significant influence on phenotypic variation of trait grain yield per square meter (Table 10). In the combined analysis of variance, the main effects, genotypes and environments were highly significant (617,250 + 1,552,340)/3,351,528 and explain 64.7% of total variation. The combined ANOVA showed grain yield of wheat was significantly affected by the environment, because significant variance at the 1% level explained 46.3% of the total variation, while the genotype contributed 18.5% of the total variation of the experiment (Table 10). 

A large sum of squares for environments indicated that the trial environments were diverse with high differences among environmental means, causing variation on grain yields of wheat. Genotype by environment interaction (GEI) expressed significant mean square, which suggests that grain yield of wheat genotypes varied across environments. The AMMI analysis revealed the complex nature of GEI and contributed 35.2% of the total sum of squares. The additional analysis of the GEI using the PCA analysis confirmed the statistical significance of the first two main components, IPCA 1 and IPCA 2 (Table 10). Separately, IPCA1 and IPCA2 participated in the GE variation with 41.2% and 29.3%, respectively, both with statistically significant effects on the GE interaction variation. These two main components jointly explained more than 70% of the variation of the genotype by environment interaction. This result is expected, since grain yield is a quantitative trait controlled by a number of minor genes and under the high influence of environmental factors.

Significant interactions between environment and wheat cultivars is in accordance with results reported by Banjac et al. [12], Popović et al. [36], Mohammadi et al. [42] and Rad et al. [51]. In addition to the influence of individual plan traits getting more important in terms of grain yield formation per area unit in stressful growing conditions, the combined analysis of variance for grain yield per m^−2^, compared with grain yield per plant trait, separated the statistical significance of the first two main components, IPCA 1 and IPCA2, which participated in the GE variation.

**Figure 3:**
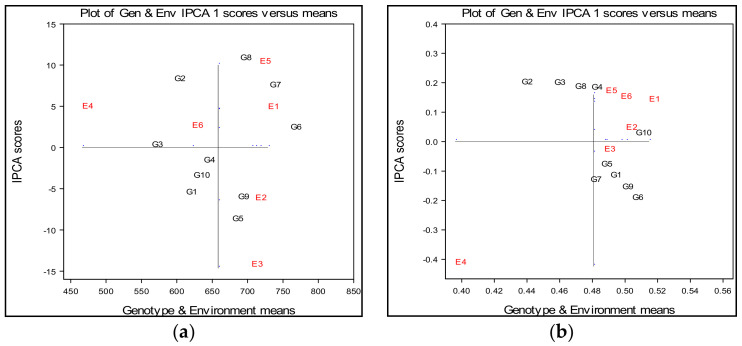
(**a**) AMMI 1 biplot of ten wheat cultivars across six environments (two years × three treatments) for the estimation of main and multivariate (GEI) effects for the grain yield in gm^−2^ at 13% moisture; (**b**) AMMI 1 biplot of ten wheat cultivars across six environments (two years × three treatments) for the estimation of main and multivariate (GEI) effects for the harvest index.

According to the biplot (Figure 3a) and in terms of the average values (Table 9), it can be observed that environment E6 exposed the lowest distance of the environmental points from the origin (zero point), which indicates that environment E6 can be assessed as the most stable environment to achieve a stable grain yield of wheat (Figure 3a).

According to the arrangement of E1, E2, E3 and E5 environments points, it can be concluded that the wheat cultivars achieved higher average values of grain yield in these environments compared to E4 and E6 points. Considering that high values of interaction indicate the poor stability of a trait, this result does not favor the environments E3 and E5 for obtaining higher values of grain yield. Environments E3 and E5 showed the highest interaction values, which determines them the least suitable for stable establishment grain yield. Regarding genotypes dispersion, genotype G3 was the most stable across all environments, indicating almost no cross interaction. The small distances from the origin were also observed for genotypes G4, G6, G10, G1, G9 and G7. Genotypes G2, G5 and G8 had a high distance from the average environment ordinate, exposed more variables and were less stable across the environments. Genotypes G6 and G7 appeared to be better adapted to the less favorable conditions of the E1 environment, keeping its average above the overall mean. This indicates that these genotypes were better adapted to stressful conditions of abiotic stress solonetz, in comparison to others cultivars. Genotypes G8 and G7 appeared to be better adapted to the conditions of the E5 environment, with its average above the overall mean, while genotypes G9 and G5 reacted favorably and responded well to melioration solonetz, since they achieved higher values of grain yield in environments E2 and E3. Genotypes G1, G2, G3, and G10 appeared to be better adapted in the E6 environment, having mean value of grain yield below the overall mean, while genotype G4 was near the overall mean (Figure 3a). According to Gauch and Moran [32], genotypes at the top of graph (such as G2, G3, G6, G7 and G8) showed positive G × E interactions with environments, while genotypes which were at the bottom (such as a G1, G4, G5, G9 and G10) showed negative G × E interactions with environments (Figure 3a).

These results are in accordance with AMMI selection of wheat genotypes across the testing environments (Table 11). Based on the tested genotypes, genotype G6 could be considered as the most promising genotype, since it ranked within first five AMMI selections in all environments, implying its best adaptability for all soil conditions. Genotypes G8, G5, G4, G2 and G6 were better adapted to stressful conditions of abiotic stress solonetz in the first growing season, while in the second season genotypes G6, G7, G9, G8 and G1 were better adapted.

Genotypes G5, G6, G4, G9 and G7 responded well to melioration solonetz in the amount of 25 tha^−1^ in the first season, while in the second season, at the same level of repair, genotypes G7, G6, G8, G9 and G2 reacted positively. Genotypes G9, G5, G6, G1 and 10 responded well to melioration solonetz in the amount of 50 tha^−1^. In the second season, at the same level of repair, genotypes G6, G7, G8, G9 and G5 reacted positively to the reparation of soil.

### 3.4. Harvest Index

Grain weight per spike plays a significant role in the yield formation, because it directly affects harvest index [46]. Increased yields in winter wheat cultivars have been found to be largely attributable to improved partitioning of biomass to the grain (i.e., higher harvest index). However, there is a biological upper limit to harvest index and; therefore, breeders need to exploit increased biomass production as the mechanism by which yields are increased [60]. Harvest index, as a measure of reproductive efficiency, presents the ratio of grain to total shoot dry matter. It has been determined by interactions between genotypes, environment and crop management [61].

The presented results showed that the mean values of harvest index ranged within different environments between 0.49% (E3) and 0.51% (E1) in the first vegetation season. In the second vegetation season, the mean values of harvest index ranged between 0.40% (E4) and 0.50% (E6) within different environments. Between genotypes, the mean values of harvest index ranged from 0.44% (G2) to 0.51% (G6 and G10) within different genotypes (Table 12). At the control variant (E1), in the first vegetation season, soil with no amelioration applied, the greatest overall mean values for grain yield were denoted for wheat varieties G10 (0.54%), G4 (0.54%), G8 (0.53%) and G6 (0.51%). In the second season at the control variant (E4), the greatest overall mean values were denoted for genotypes G6 (0.51%), G9 (0.48%) and G7 (0.54%), Table 12.

In the second environment, with soil amelioration of 25 tha^−1^ phosphor gypsum applied, during the first vegetation (E2), the greatest overall mean values for harvest index were denoted for wheat varieties G10 (0.54%), G9 (0.51%), G5 (0.51%) and G1(0.51%). In the second season, with same level of amelioration (E5), the greatest overall mean values were denoted for wheat varieties G10 (0.52%), G8 (0.50%), G4 (0.51%) and G6 (0.48%), Table 12.

In the third environment during the first vegetation season at the amelioration of 50 tha^−1^ phosphor gypsum applied (E3), the greatest overall mean values for harvest index were denoted for wheat varieties G6 (0.52%), G9 (0.51%), G10 (0.51%) and G1 (0.50%). In the second season, with same level of amelioration (E6), the greatest overall mean values of harvest index were denoted for wheat varieties G10 (0.53%), G4 (0.52%), G8 (0.51%), G6 (0.49%) and G3 (0.50). Presented results revealed that different treatments influenced the differences in grain weight per plant (Table 12).

According to the mean values of both seasons, higher sensitivity of harvest index could be observed in the second growing season, as the environmental average was the lowest in the control variant of soil, without ameliorative measures, as a consequence of variable conditions of the 2005/2006 vegetation period. On the other side, more favorable conditions during the first vegetation season showed significant impact on reducing differences between the mean values per treatment and indicated that the effect of a particular soil treatment was missing. Therefore, the changing weather conditions highly influenced the effect of soil melioration on the harvest index and other grain yield traits of wheat. Since each vegetation season could bring certain differences due to even more unpredictable weather conditions, cultivars’ assessment across several years is more reliable and could bring appropriate recommendation of cultivars characterized by a small error.

The combined ANOVA showed that all three sources of variation (genotypes, treatments and environment) were statistically significant, having significant influence on phenotypic variation of the harvest index. In the combined analysis of variance, the main effects, genotypes and environments were highly significant and explained 60.6% of the total variation. Participation of the genotype variation in the treatment sum of squares (SS) amounted to 13.4%, while 47.1% of the total sum of squares was attributable to environmental effects (Table 13). A large sum of squares for environments indicated the differences between growing seasons, while diversity of treatments caused a considerable sum of squares for environmental factors in the total variation, which indicates that these factors were the most responsible for the variation of the harvest index. Genotype by environment interaction expressed a significant mean square which represented 39.4%. Sum of squares of GEI was 2.9 times higher than the sum of the squares of genotype, indicating that there were significant differences in behavior between genotypes in different environments. Additional analysis of the GEI using the PCA analysis showed a statistical significance of the first source of variation, first main component IPCA1, which participated in the GEI variation with 78.54%. In addition to the two main components jointly explaining more than 90% of the GEI variation, the second source of variation, the second main component IPCA2, expressed no statistically significant effect in the GE interaction variation (Table 13). Significant interactions between environment and wheat cultivars in the harvest index, as a high share of the first main component IPCA1 in the GE variation, have been reported by Dimitrijević et al. [10]. However, a higher influence of genetic factor on the harvest index of wheat was observed by Tayar [62].

According to the biplot (Figure 3b) and in terms of average values (Table 13), it could be noticed that, apart from certain exceptions, agro-ecological environments were quite near the level of the experimental overall average. A small distance of the environmental points from the origin (zero point) indicates that the environments E3 and E2 could be assessed as the most favorable to achieve a stable response of harvest index of wheat. Environments E5, E6 and E1 could be assessed as less stable, while environment E4 had the highest negative interaction value, which determines them the least suitable for the stable establishment harvest index of wheat. Wheat cultivars G1, G5, G6, G7 and G9 achieved higher average values of harvest index in E3 environment, while genotype G10 achieved the greatest value of harvest index in E2 environment. In general, wheat variety G10 (Simonida) expressed the most stable reaction for this trait, showing a PCA1 score close to 0, and it responded well when grown in E2 environment, within soil amelioration of 25 t ha^−1^ phosphogypsum applied, (Figure 3b). Wheat genotypes G3, G4, G8 and at least G2 responded favorably within treatments of 50 tha^−1^ phosphogypsum applied. According to Gauch and Moran [32], G × E interactions are estimated based on the position of wheat genotypes within the graph. Therefore, genotypes at the top of graph (such as G2, G3, G4, G8 and G10) showed positive G × E interactions with environments, while genotypes which were at the bottom (such as a G1, G5, G6, G7 and G9) showed negative G × E interactions with environments (Figure 3b). However, in addition to showing the most stable reaction, genotype G10 achieved the greatest mean value of harvest index. In this analysis, wheat genotype G6 also expresses the greatest value of harvest index, but followed with high interaction values, which indicates it as the least suitable for the stable establishment harvest index. Wheat varieties with improved harvest index are desirable since they expressed increased physiological efficiency of nutrient reutilization and efficiency in translocation of nutrients from leaves and stems into grains.

These results are in accordance with AMMI selection of wheat genotypes across the testing environments (Table 14). 

Results of the harvest index of the environments and their respective scores on the first axis of GEI showed similar results as previous analysis. In the first vegetation season, in environment E1 (control, soil without amelioration), genotype G10 could be considered as the most promising genotype, since it ranked first, followed with genotypes G4, G8 and G6. In soil environment E4 (control without amelioration) in the second vegetation season, genotype G6 was first ranked, followed by genotypes G9, G7 and G1. These results indicate that these genotypes showed positive response and stable reaction in less favorable conditions on control environments. In soil environment E2 (treatment with 25 tha^−1^ phosphogypsum), in the first vegetation season, genotype G9 was first ranked, implying its best adaptability for this soil conditions, followed by genotypes G5, G1 and G10. In soil environment E5 (treatment with 25 tha^−1^ phosphogypsum), in the second vegetation season, genotype G10 was first ranked, followed by genotypes G8, G4 and G6, which indicates that these genotypes showed positive response on soil melioration treatment with 25 tha^−1^ phosphogypsum. In soil environment E3 (treatment with 50 tha^−1^ phosphogypsum), in the first vegetation season, genotype G9 could be the most promising genotype, since it ranked first, followed by genotypes G5, G1 and G10. In soil environment E6 (treatment with 50 tha^−1^ phosphogypsum), in the second season, genotype G10 was first ranked, followed by genotypes G4, G8 and G6, which indicates that these genotypes showed positive response on soil amelioration with amount of 50 tha^−1^ phosphogypsum. These results support the recommendation of appropriate genotype for all environments, or specific genotypes for specific environments, through AMMI evaluation, Table 14.

According to the results, a greater sensitivity of harvest index was observed in less favorable conditions in the second vegetation period. In the second vegetation period, the general mean value of the harvest index was the lowest in the control variant, without ameliorative measures. More favorable conditions in the first vegetation season had a significant impact on reducing the differences between the averages values per treatment. This result is expected, since the harvest index is a highly variable trait and depends on the weather conditions during the season. Therefore, in a more favorable year the harvest index is higher. Since the harvest index represents efficiency of translocation of the nutrients of plants from vegetative to generative parts, and this trait highly depends on two pronounced quantitative traits, grain weight per plant and plant biomass, it is a highly variable trait. The similar results and high impact of year on this trait were observed by Vuković [63] and Kondic [64]. The results suggested that the harvest index is a critical factor for grain yield of wheat under variable weather conditions, terminal high temperatures, as well as in water shortages.

In general, obtained results indicated that even in wheat, which is considered to be durable and well adapted to a wide range of environments, in addition to the influence of different treatments, different climatic conditions can also cause significant differences in some important yield related traits. Furthermore, variable genotype reactions to different environments point out the importance of the evaluation of different wheat cultivars in order to identify and recommend the optimal genotype for specific growing conditions [64,65,66].

## 4. Conclusions

Based on the presented findings it can be concluded that the application of complex ameliorative measures could increase the production potential of the solonetz soil near to a level of productive soils. The results also indicated that amelioration soil measures, with adequate selection of appropriate wheat genotypes and adequate management practices, could mean that these soils could provide high and stable wheat yields. Differences in growing seasons contributed considerably to treatment effects. The more favorable weather conditions of the first season exhibited a more significant impact on minimizing the differences between mean values per treatment and; therefore, the greatest overall stability was evident in the first vegetation season.

Based on the tested genotypes, genotype G6 (Evropa 90) expressed a stable reaction over all environments for the traits plant height, grain weight per plant and grain yield per square meter. In addition to this stable reaction, this genotype resulted in greater values of observed traits and could be considered as one of the most promising genotypes in all soil environments. The greater expression for both plant height and grain yield of genotype in all soil conditions could be attributed to the high capacity of the stem to accumulate sufficient stem reserves for the partitioning to grain. This indicates that the genotypes that could maintain their height in stress environments could be desirable. However, genotype G10 (Simonida) could be considered as a wheat variety with improved harvest index, since it expressed the most stable reaction for this trait, showing a PCA1 score close to 0, followed with the greatest mean value of the harvest index. On the other side, genotype G6 (Evropa 90), which showed also the greatest mean values for the harvest index, would be recommended if the GEI was disregarded. However, high yielding, but not stable wheat genotypes across environments could be recommended the specific environments where they performed well. Information in this regard would help wheat producers, as well as breeders, to select the appropriate wheat cultivars that can be successfully exploited in conditions of less productive soils, such as solonetz type.

## Figures and Tables

**Table 1:** Description of the six different environments used to evaluate ten winter wheat genotypes.

Environments Code	Growing Seasons	Treatments by Phosphogypsum
E1	2004/2005	Control–non-ameliorated solonetz
E2	2004/2005	Solonetz ameliorated by 25 tha^−1^ phosphogypsum
E3	2004/2005	Solonetz ameliorated by 50 tha^−1^ phosphogypsum
E4	2005/2006	Control–non-ameliorated solonetz–control
E5	2005/2006	Solonetz ameliorated by 25 tha^−1^ phosphogypsum
E6	2005/2006	Solonetz ameliorated by 50 tha^−1^ phosphogypsum

**Table 2:** Chemical composition of solonetz soil.

Parameter	pH		CaCO_3_	Humus	Total N	P_2_O_5_	K_2_O	Salt
Depth (cm)	KCl	H_2_O	(%)	(%)	(%)	mg/100 g Soil		(%)
0–10	4.50	5.80	0.00	5.95	0.40	7.20	85.00	0.03
11–30	6.30	7.85	0.26	1.68	0.11	1.60	17.50	0.13
31–60	6.90	8.20	0.02	1.47	0.07	5.30	23.40	0.18

**Table 3:** Mean values for the trait plant height of wheat (cm) and interaction IPCA1 values of the AMMI model of ten winter wheat varieties grown in six environments.

Plant Height (cm)													
Genotypes (G)													
E	G1	G2	G3	G4	G5	G6	G7	G8	G9	G10	Em	IPCAe1	V
E1	61.0	59.9	60.3	66.9	65.6	68.6	65.5	64.6	63.9	59.3	63.5	1.184	73.4
E2	61.3	60.0	59.8	66.4	64.1	68.5	64.9	65.2	63.7	60.9	63.5	0.528	42.0
E3	60.3	58.9	58.0	64.5	61.2	67.1	63.0	64.7	62.2	61.5	62.1	−0.209	86.8
E4	39.7	38.7	40.2	47.0	47.1	47.8	45.5	42.6	43.3	35.7	42.8	2.287	67.2
E5	57.5	56.0	53.3	59.6	54.0	63.5	58.1	63.0	58.2	62.4	58.6	−1.952	39.6
E6	47.2	45.7	43.2	49.5	44.0	53.3	48.0	52.6	48.0	51.9	48.3	−1.838	57.6
IPCAg1	0.4688	−0.3468	0.6165	0.7413	2.1104	0.0333	0.7631	−1.0834	0.2071	−2.572	56.5	1.184	124.7
Gm	54.5	53.2	52.4	59.0	56.0	61.5	57.5	58.8	56.5	55.3			

AMMI: The additive main effects and multiplicative interaction; Gm: Genotype mean; Em: Environmental means; IPCAe1: The first interaction principal component axes for environment; IPCAg1: Interaction principal component axes for genotype; G: wheat genotypes; E: Environmental labels with control (K) and 25 or 50 tha^−1^ phosphogypsum applied in both seasons; V: Variance.

**Table 4:** AMMI analysis of variance for the plant height of ten winter wheat varieties examined across six environments.

Source ^1^	df	SS	MS	F-Value	F-prob	The Share of Total Variation %
Total	179	22,318	124.7	-	-	
Treatments	59	14,588	247.3	4.81 **	0.0000	65.36
Genotypes	9	1260	140	2.72 **	0.0067	8.64
Environments	5	11,691	2338.1	12.89 **	0.0000	80.14
Block	12	2176	181.3	3.53 **	0.0002	14.92
Interactions	45	1638	36.4	0.71 ^ns^	0.9039	11.23
IPCA1	13	600	46.2	0.90 ^ns^	0.5579	36.63
IPCA2	11	476	43.3	0.84 ^ns^	0.5992	29.06
Residuals	21	562	26.7	0.52 ^ns^	0.9567	3.85
Error	108	5553	51.4	-	-	-

^1^ All sources were tested in relation to the error; **: Highly significant at *p* < 0.01 probability level; ^ns^: Not significant; df: Degree of freedom; F: F value calculated; IPCA1: The first interaction principal components axes; IPCA2: The second interaction principal components axes.

**Table 5:** Table of first AMMI selections per environment.

No.	E	Mean	IPCA	Genotypic Rank									
		(g)	Score	1	2	3	4	5	6	7	8	9	10
4	E4	42.8	2.287	G6	G4	G5	G7	G8	G9	G1	G3	G2	G10
1	E1	63.5	1.184	G6	G4	G8	G7	G5	G9	G1	G10	G2	G3
2	E2	63.5	0.528	G6	G8	G4	G7	G9	G10	G5	G1	G2	G3
3	E3	62.1	−0.209	G6	G5	G4	G7	G9	G8	G3	G1	G2	G10
6	E6	48.3	−1.838	G6	G8	G10	G4	G9	G7	G1	G2	G5	G3
5	E5	58.6	−1.952	G6	G8	G10	G4	G9	G7	G1	G2	G5	G3

E: Environments; IPCA score: Score based on the first interaction principal component axes.

**Table 6:** Mean values for the grain weight per plant of wheat (g) and interaction PCA1 values of the AMMI model of ten winter wheat varieties grown in six environments.

Grain Weight Per Plant (g)													
Genotypes (G)													
E	G1	G2	G3	G4	G5	G6	G7	G8	G9	G10	Em	IPCAe1	V
E1	5.78	6.13	5.69	6.28	6.41	7.53	7.44	7.15	6.59	6.10	6.51	0.3463	2.0
E2	6.24	5.35	5.55	6.32	7.09	7.08	6.58	6.05	6.97	6.28	6.35	−0.6171	1.4
E3	6.67	4.78	5.50	6.41	7.70	6.78	5.96	5.23	7.35	6.49	6.29	−1.3967	2.7
E4	3.20	3.78	3.22	3.78	3.79	5.11	5.10	4.86	4.02	3.57	4.04	0.5236	3.3
E5	5.31	6.40	5.57	6.06	5.80	7.59	7.74	7.61	6.15	5.79	6.40	0.9248	1.4
E6	4.90	5.09	4.73	5.35	5.56	6.53	6.38	6.38	5.70	5.18	5.55	0.2192	1.4
IPCAg1	−0.636	0.6512	−0.017	−0.201	−0.870	0.297	0.718	0.975	−0.566	−0.351			2.7
Gm	5.35	5.25	5.04	5.70	6.06	6.77	6.53	6.16	6.13	5.57			

Gm: Genotype mean; Em: Environmental means; IPCAe1: The first interaction principal component axes for environment; IPCAg1: Interaction principal component axes for genotype; E: Environmental labels with control (K) and 25 or 50 tha^−1^ phosphogypsum applied in both seasons; V: variance.

**Table 7:** AMMI analysis of variance for the grain weight per plant (g) of ten winter wheat varieties examined across six environments.

Source ^1^	df	SS	MS	F-Value	F-Prob	The Share of Total Variation %
Total	179	488.4	2.728	-	-	-
Treatments	59	284.1	4.816	3.04 **	0.0000	58.17
Genotypes	9	52	5.773	3.64 **	0.0005	18.30
Environments	5	136.1	27.23	9.85 **	0.0000	48.97
Block	12	33.2	2.765	1.75 *	0.0669	11.69
Interactions	45	96	2.134	1.34	0.1074	33.79
IPCA 1	13	39.5	3.039	1.92 *	0.0355	41.15
IPCA 2	11	27.1	2.46	1.55	0.1234	28.23
Residuals	21	29.5	1.403	0.89	0.6093	10.38
Error	108	171.1	1.584	-	-	-

^1^ All sources were tested in relation to the error; *: Significant at *p* < 0.05 probability level; **: Highly significant at *p* < 0.01 probability level; df: Degree of freedom; F: F value calculated; IPCA: Interaction principal components axes.

**Table 8:** Table of first AMMI selections per environment.

No.	E	Mean	IPCA	Genotypic Rank									
		(g)	Score	1	2	3	4	5	6	7	8	9	10
5	E5	6.40	0.9248	G7	G8	G6	G2	G9	G4	G5	G10	G3	G1
4	E4	4.04	0.5236	G6	G7	G8	G9	G5	G2	G4	G10	G3	G1
1	E1	6.51	0.3463	G6	G7	G8	G9	G5	G4	G2	G10	G1	G3
6	E6	5.55	0.2192	G6	G7	G8	G9	G5	G4	G10	G2	G1	G3
2	E2	6.35	−0.6171	G5	G6	G9	G7	G4	G10	G1	G8	G3	G2
3	E3	6.29	−1.3967	G5	G9	G6	G1	G10	G4	G7	G3	G8	G2

E: Environments; IPCA score: Score based on the first interaction principal component axes.

**Table 9:** Mean values for the grain yield of wheat expressed in gm^−2^ at 13% moisture and interaction PCA1 values of the AMMI model of ten winter wheat varieties grown in six environments.

Grain Yield Per m^2^ (g)													
Genotypes (G)													
E	G1	G2	G3	G4	G5	G6	G7	G8	G9	G10	Em	IPCAe1	V
E1	580.90	780.70	704.00	785.90	816.50	762.50	749.20	819.90	623.50	663.70	728.7	4.4755	20,653
E2	671.70	631.00	647.60	740.80	837.20	767.00	701.50	679.60	736.90	696.00	710.9	−6.5731	17,808
E3	772.10	502.10	590.10	685.60	822.30	804.80	703.50	580.30	863.50	731.70	705.6	−14.638	30,580
E4	448.00	384.10	322.60	375.70	367.90	632.60	631.50	535.80	538.90	423.30	466.0	4.5323	34,238
E5	638.10	708.10	599.50	648.60	610.40	865.30	889.40	848.80	715.10	648.40	717.2	10.0010	15,810
E6	566.70	573.20	524.20	592.90	616.40	731.90	714.30	675.40	640.20	578.90	621.4	2.2030	15,536
IPCAg1	−5.8809	7.8640	−0.1107	−1.9985	−9.1038	2.0113	7.1011	10.4235	−6.4573	−3.8487	658.3	0.0000	30,483
Gm	612.90	596.50	564.70	638.20	678.40	760.70	731.60	690.00	686.30	623.70			

Gm: Genotype mean; Em: Environmental means; IPCAe1: The first interaction principal component axes for environment; IPCAg1: Interaction principal component axes for genotype; E: Environmental labels with control (K) and 25 or 50 tha^−1^ phosphogypsum applied in both seasons; V: Variance.

**Table 10:** AMMI analysis of variance for the grain yield of ten winter wheat varieties in gm^−2^ at 13% moisture examined across six environments.

Source ^1^	df	SS	MS	F-Value	F-prob	The Share of Total Variation %
Total	179	5,456,441	30,483	*	*	
Treatments	59	3,351,528	56,806	3.60 **	0.0000	61.40
Genotypes	9	617,250	68,583	4.35 **	0.0001	18.45
Environments	5	1,552,340	310,468	9.26 **	0.0000	46.30
Block	12	402,215	33,518	2.13 *	0.0208	12.00
Interactions	45	1,181,937	26,265	1.67 **	0.0168	35.20
IPCA1	13	487,088	37,468	2.38 **	0.0077	41.21
IPCA2	11	346,349	31,486	2.00 *	0.0354	29.30
Residuals	21	348,501	16,595	1.05	0.4098	10.40
Error	108	1,702,698	15,766	*	*	-

^1^ All sources were tested in relation to the error; *: Significant at *p* < 0.05 probability level; **: Highly significant at *p* < 0.01 probability level; df: Degree of freedom; F: F value calculated; IPCA1: The first interaction principal components axes; IPCA2: The second interaction principal components axes.

**Table 11:** Table of first AMMI selections per environment.

No.	E	Mean	IPCA	Genotypic Rank									
		(g)	Score	1	2	3	4	5	6	7	8	9	10
5	E5	717.20	10.0010	G7	G6	G8	G9	G2	G4	G10	G1	G5	G3
4	E4	466.00	4.5320	G6	G7	G9	G8	G1	G10	G2	G4	G5	G3
1	E1	728.70	4.4760	G8	G5	G4	G2	G6	G7	G3	G10	G9	G1
6	E6	621.40	2.2030	G6	G7	G8	G9	G5	G4	G10	G2	G1	G3
2	E2	710.90	−6.5730	G5	G6	G4	G9	G7	G10	G8	G1	G3	G2
3	E3	705.60	−14.6390	G9	G5	G6	G1	G10	G7	G4	G3	G8	G2

E: Environments; IPCA score: Score based on the first interaction principal component axes.

**Table 12:** Mean values for harvest index (%) and interaction PCA1 values of the AMMI model of ten wheat varieties grown in six environments.

Harvest Index (%)													
Genotypes (G)													
E	G1	G2	G3	G4	G5	G6	G7	G8	G9	G10	Em	IPCAe1	V
E1	0.51	0.50	0.52	0.54	0.51	0.51	0.49	0.53	0.51	0.54	0.51	0.1303	0.001
E2	0.51	0.46	0.48	0.51	0.51	0.52	0.49	0.50	0.51	0.53	0.50	0.0342	0.003
E3	0.50	0.44	0.46	0.48	0.50	0.52	0.49	0.47	0.51	0.51	0.49	−0.0395	0.003
E4	0.46	0.27	0.29	0.32	0.44	0.51	0.45	0.31	0.48	0.42	0.40	−0.4241	0.021
E5	0.48	0.47	0.49	0.51	0.48	0.48	0.46	0.50	0.48	0.52	0.49	0.1592	0.002
E6	0.49	0.48	0.50	0.52	0.49	0.49	0.48	0.51	0.49	0.53	0.50	0.1399	0.002
PCAg1	−0.127	0.188	0.1867	0.1703	−0.0910	−0.2039	−0.1433	0.1728	−0.168	0.0153	0.48		0.007
Gm	0.49	0.44	0.46	0.48	0.49	0.51	0.48	0.47	0.49	0.51			

Gm: Genotype mean; Em: Environmental means; IPCAe1: The first interaction principal component axes for environment; IPCAg1: Interaction principal component axes for genotype; E: Environmental labels with control (K) and 25 or 50 tha^−1^ phosphogypsum applied in both seasons; V: Variance.

**Table 13:** AMMI analysis of variance for the harvest index (%) of ten winter wheat varieties examined across six environments.

Source ^1^	df	SS	MS	F-Value	F-prob	The Share of Total Variation %
Total	179	1.196	0.00668	*	*	
Treatments	59	0.5794	0.00982	2.59 **	0.00001	48.44
Genotypes	9	0.0779	0.00866	2.28 *	0.02199	13.44
Environments	5	0.2732	0.05463	3.17 **	0.01043	47.15
Block	12	0.2069	0.01724	4.55 **	0.00001	35.71
Interactions	45	0.2283	0.00507	1.35 *	0.11293	39.40
IPCA 1	13	0.1793	0.01379	3.64 **	0.00009	78.54
IPCA 2	11	0.0281	0.00255	0.67	0.76147	12.31
Residuals	21	0.0209	0.00100	0.26	0.99953	3.61
Error	108	0.4097	0.00379	*	*	

^1^ All sources were tested in relation to the error; *: Significant at *p* < 0.05 probability level; **: Highly significant at *p* < 0.01 probability level; df: Degree of freedom; F: F value calculated; IPCA: Interaction principal components axes.

**Table 14:** Table of first AMMI selections per environment.

Number	E	Mean	IPCA	Genotypic Rank									
		(%)	Score	1	2	3	4	5	6	7	8	9	10
5	E5	0.4883	0.1592	G10	G8	G4	G6	G7	G2	G3	G1	G9	G5
6	E6	0.4973	0.1399	G10	G4	G8	G6	G3	G9	G1	G5	G2	G7
1	E1	0.5147	0.1303	G10	G4	G8	G6	G3	G9	G1	G5	G7	G2
2	E2	0.5007	0.0343	G9	G5	G1	G10	G4	G3	G6	G8	G7	G2
3	E3	0.4873	−0.0396	G9	G5	G1	G10	G6	G4	G3	G7	G8	G2
4	E4	0.396	−0.4241	G6	G9	G7	G1	G5	G10	G4	G8	G3	G2

E: Environments; IPCA score: Score based on the first interaction principal component axes.
